# Hantaviruses in Agricultural and Forestry Workers: Knowledge, Attitudes and Practices in Italian Physicians

**DOI:** 10.3390/tropicalmed6030169

**Published:** 2021-09-20

**Authors:** Matteo Riccò, Pietro Ferraro, Simona Peruzzi, Federica Balzarini, Silvia Ranzieri

**Affiliations:** 1 Servizio di Prevenzione e Sicurezza Negli Ambienti di Lavoro (SPSAL), AUSL-IRCCS di Reggio Emilia, Via Amendola n.2, I-42122 Reggio Emilia, RE, Italy; 2 Department of Prevention, Occupational Health and Safety Service of the Local Health Unit of Foggia, ASL Foggia, Piazza Pavoncelli 11, I-41121 Foggia, FG, Italy; dott.pietro.ferraro@gmail.com; 3 Laboratorio Analisi Chimico Cliniche e Microbiologiche, Ospedale Civile di Guastalla, AUSL-IRCCS di Reggio Emilia, I-42016 Guastalla, RE, Italy; simona.peruzzi@ausl.re.it; 4 Dipartimento P.A.A.P.S.S., Servizio Autorizzazione e Accreditamento, Agenzia di Tutela della Salute (ATS) di Bergamo, Via Galliccioli, 4, I-24121 Bergamo, BG, Italy; federica.balzarini@ats-bg.it; 5 Department of Medicine and Surgery, University of Parma, Via Gramsci, 14, I-43126 Parma, PR, Italy; silvia.ranzieri@unipr.it

**Keywords:** hantaviruses, knowledge, risk perception, hantavirus cardiopulmonary syndrome, hantavirus pulmonary syndrome, health knowledge, practice, vaccines

## Abstract

Hantaviruses are viral pathogens usually endemic in rodent populations. Human exposure follows inhalation of dusts contaminated with rodent excreta, and most individuals have been infected in occupational settings heavily contaminated with rodent droppings, such as agricultural and forestry. To date, knowledge, attitudes and practices of medical professionals, especially occupational physicians (OP), regarding hantavirus disease in at-risk workers have been scarcely investigated. We investigated these topics through a structured questionnaire administered through an online survey of 223 medical professionals (42.2% of them working as OP). Adequate general knowledge of hantavirus disease was found in 48.9% of respondents, with OP exhibiting a better understanding of clinical features of human hantavirus infections. OP aware of the endemic status of hantavirus in North-Eastern Italy exhibited higher risk perception for agricultural workers (odds ratio 21,193, 95% confidence interval 3.666–122.505). On the contrary, a better knowledge of hantaviruses was association with acknowledging an increased risk of hantavirus infection in forestry workers (odds ratio 5.880, 95% confidence interval 1.620–21.343). Hantavirus in Italy represent an often-overlooked biological risk in occupational settings. The lack of preventive immunization, the inappropriate risk perception and the unsatisfying awareness of hantavirus issues collectively stress the importance of appropriate information campaigns among health care providers.

## 1. Introduction

Hantaviruses (family *Hantaviridae*) are monopartite, trisegmented, negative-stranded enveloped RNA viruses belonging to the order of *Bunyavirales* [1,2,3]. Usually dichotomized as Old World (or Eurasian), and New World (or American) pathogens, hantaviruses can cause different clinical syndromes [1,4,5]. As seroprevalence estimates usually exceed official reports on human infections, most of them occur unnoticed, usually as mild flu-like syndrome, sometimes characterized by high fever, malaise and myalgia, and only a variable share of incident cases develops severe systemic disorders, with high mortality rates [1,2]. For example, the most frequently reported European hantavirus, the Puumala virus (PUUV), is usually associated with a mild clinical syndrome known as “nephropathia epidemica” (NE), which has a low case fatality rate of approximately 0.4% [6]. East Asian (e.g., Hantan virus and Seoul virus) and the European Dobrava-Belgrade virus (DOBV) more frequently cause a severe disease with renal failure and hemorrhagic manifestations varying from petechiae to internal bleedings (hemorrhagic fever with renal syndrome, HFRS), and a case fatality rate up to 15% [6,7]. With 100,000 to 200,000 incident cases every year, HFRS largely exceeds the burden of disease associated with American hantaviruses (e.g., Andes virus and Sin Nombre virus), causes of a severe syndrome characterized by pneumonia and cardiopulmonary disfunction (i.e., hantavirus cardiopulmonary syndrome, or HCPS), whose case fatality rate may range up to 40%. The striking heterogeneity of hantaviruses is a consequence of their co-evolution with the usual hosts, mainly rodents and insectivores [3], but also chiropters [1,8]. In fact, inter-human spreading is substantially unlikely [1], being documented only with the Andes virus, and human infections represent a substantial “cul-de-sac” that follow the inhalation of fomites from an infected host (i.e., urine, feces, saliva or contaminated dusts) [1,2,3].

As the main risk factor for hantavirus infections is represented by human-host (i.e., rodent) contact, such pathogens may represent an occupational risk agent for a broad array of professionals: veterinarians, laboratory scientists and technicians, military personnel, forestry workers and farmers [1]. Not coincidentally, studies performed on high-risk occupational groups from Western and North-European countries such as Spain [9], Germany [10,11], Netherlands [12], Hungary [13], Slovakia [14], Sweden [15,16] and Norway [17] have reported seroprevalence rates ranging from 3.0 to 9.1%, peaking to 30.6% in some selected subgroups, significantly exceeding those of the general population of the parent country.

Despite its proximity to endemic countries (e.g., Slovenia, Austria and Germany), according to available statistics from the European Centre for Disease Prevention and Control (ECDC), to date no autochthonous cases have been officially reported in Italy [18]. However, previous studies from the Italian Cadore area (a historical region in the Italian region of Veneto, in the mountainous northernmost part of the province of Belluno bordering on Austria, the Trentino-Alto Adige/Süd Tirol and Friuli-Venezia Giulia) [19,20,21,22], and from the nearby Trentino Alto-Adige [23,24,25], have reported a hantavirus seroprevalence of 4.0% (range 1.3–11.7%) for forestry workers, and 5.3% (3.5–8.0%) for local farmers. In other words, even though hantavirus infections are not properly diagnosed by the responsible medical professionals, including the competent occupational physicians (OP), and mostly not notified, human infections do occur, most often in these occupational groups. In fact, hantaviruses are mostly perceived as uncommon pathogens, with subsequent diagnostic delays or even misdiagnoses [22,25,26]. As a consequence, the assessment of specific knowledge (i.e., the awareness of the health threat, in this case represented by hantaviruses), attitudes (i.e., propensity towards a certain intervention) and practices (i.e., actual application of such intervention; collectively, KAP)—of Italian physicians, and particularly of OP, the medical professionals responsible for health surveillance and promotion in workplace [27], can be useful in order to improve the health and safety of high-risk outdoor workers. 

Therefore, this study's objective is to assess the KAP of Italian physicians (especially OPs) about human hantavirus infections. This study will be assessing which factors are most often associated with having a better understanding of hantaviruses as a potential occupational health threat in Italy. In fact, an appropriate identification and investigation of these factors can actively contribute to prevention and control programs, by designing interventions aimed to improve the overall awareness of medical professionals about these pathogens and their clinical consequences in high-risk groups.

## 2. Materials and Methods

### 2.1. Study Design

A cross-sectional questionnaire-based study was performed between 1 August 2020 and 31 August 2020, involving Italian physicians participating in nine different private Facebook group pages, and four closed forums on general medicine. In both cases, applications were officially limited to registered medical professionals. A total of approximately 4043 unique members was eventually reached, but no information could be obtained regarding cross-inscriptions, not even how many of these members were actively using the parent platform at the time of the survey.

To post the study invitation, the chief researcher contacted the administrators, requesting preventive authorization to post the link to the questionnaire, including a short description of the aims of the survey. Users who clicked on the invitation texts were provided with the full study information, an opportunity to give their informed consent, and a web link to the survey (Google Forms; Google LLC; Menlo Park, California, CA, USA). The survey was conducted in Italian. To be included in the sample, the participant was supposed to be living and working in Italy as a medical professional. If a potential participant was found not to match the inclusion criteria, the survey closed down. The survey was anonymous, and no personal data, such as name, IP address, email address or personal information unnecessary to the survey, was requested, saved or tracked. No monetary or other compensation was offered to the participants.

### 2.2. Questionnaire

The test–retest reliability of the questionnaire was preventively assessed through a survey on 15 medical professionals completing the questionnaire at two different points in time. The testing questionnaires were ultimately excluded from the final analyses. All questions were self-reported, and not externally validated. An English translation of the questionnaire is available on request from the corresponding author. The final questionnaire included the following sections:

2.2.1. Individual characteristics: age, sex, Italian region of origin (i.e., where the respondent mainly works) and whether they had:

(a) Any knowledge that hantavirus infections may occur in Italy (yes vs. no);
(b) Any personal and/or professional interaction with hantavirus infections (i.e., managing of infected patient(s), personal infection, infection in friends, relatives, etc.).

2.2.2. Risk perception: participants were initially asked to rate the perceived severity (C^H^) and the perceived frequency (I^H^) of hantavirus infections in Italy by means of a fully labeled 5-points Likert scale. The available options ranged from “not significant” (i.e., “of no significant concern in daily practice”, score 1) to “very significant” (i.e., “of very high concern in daily practice”, score 5). As perceived risk has been defined as a function of the perceived probability of an event and its expected consequences [28,29], a risk perception score (RPS) was eventually calculated as follows, and reported as a percent value:

RPS = I^H^ × C^H^ (1)

2.2.3. Knowledge test: participants received a set of 10 true-false statements and 5 multiple-choice items on human hantavirus infection that were elaborated through extensive literature review (e.g., hantaviruses are characterized by frequent interhuman spreading—FALSE) [1,2,4,7,12,25,26,30,31,32,33,34,35,36]. A general knowledge score (GKS) was then calculated as the sum of correctly and incorrectly marked recommendations: when the participants answered correctly, +1 was added to a sum score, whereas a wrong indication or a missing/“don’t know” answer added 0 to the sum score. GKS was then dichotomized by median value in higher vs. lower knowledge status. A series of symptoms were then reported to the participants, and they were asked to report which ones they perceived as usually associated with Old World hantavirus infections (i.e., fever > 38 °C, headache; abdominal pain; back pain; nausea; petechiae; hypotension; oliguria (i.e., <0.5 L/24 h); polyuria (i.e., >2.0 L/24 h); leukocytosis (i.e., count of white blood cells >10 × 10^9^/L); thrombocytopenia (i.e., platelet count 52–75 × 10^9^/L); proteinuria; hematuria).

2.2.4. Attitudes and Practices: we inquired participants about the perceived risk for human hantavirus infections compared to the general population in five occupational groups, i.e., 3 mainly outdoor workers (i.e., forestry workers, agricultural workers and construction workers), a group of “indoor” workers (i.e., food processing workers) and workers actively interacting with large animals (i.e., dairy farmers). Perceived risk was rated by participants by means of a fully labeled 5-point Likert scale, whose available options ranged from “totally disagree” to “totally agree”. Reported answers were ultimately dichotomized in somewhat agree (i.e., “agree” and “totally agree”) vs. somewhat disagree (i.e., “neutral”, “disagree” and “totally disagree”).

### 2.3. Data Analysis

Continuous variables were initially tested for normal distribution (D’Agostino and Pearson omnibus normality test): where the corresponding *p* value was < 0.10, “normal” distribution was assumed as rejected, and variables were compared through Mann–Whitney or Kruskal–Wallis tests for multiple independent samples. On the other hand, variables passing the normality check (D’Agostino and Pearson *p* value ≥ 0.10) were compared using the Student’s *t* test or ANOVA, where appropriate. Categorical variables were reported as per cent values, and their distributions in respect of the outcome variables of agreeing towards an increased risk of hantavirus infections in farmers and forestry workers were initially analyzed through chi-squared test. 

All categorical variables that at univariate analysis were associated with the aforementioned statuses with a *p* value < 0.25 were included as explanatory variables in a stepwise binary logistic regression analysis model having risk perception in farmers and forestry workers as outcome variables. Adjusted odds ratios (aOR) and their respective 95% confidence intervals (95% CI) were calculated accordingly. All statistical analyses were performed by means of IBM SPSS Statistics 25.0 for Macintosh (IBM Corp. Armonk, NY, USA) and R 4.0.3 [37] by means of packages epiR (v. 2.0.19), EpiReport (v 1.0.1), fmsb (0.7.0), plot3d (1.3), msm (1.6.8) and sandwich (3.0–0).

### 2.4. Ethical Considerations

Before giving their consent to the survey, participants were briefed that all information would be gathered anonymously and handled confidentially. Participation was voluntary, and the questionnaire was collected only from subjects who had expressed consent for study participation. Identification of individual participants by means of the presented material is impaired by the lack of personal data such as the community of residence, the precise occupational setting, etc. Because of the anonymous, observational design, the lack of clinical data about patients, as the study did not configure itself as a clinical trial, a preliminary evaluation by an Ethical Committee was not required, according to the Italian law (Gazzetta Ufficiale no. 76, dated 31 March 2008).

## 3. Results

### 3.1. Descriptive Analysis: General Characteristics of the Sample

As shown in Table 1, a total of 223 Italian physicians out of the 4043 eligible professionals (5.5%) participated into the inquiry. The mean age of the respondents was 44.2 ± 8.2 years, and 42.6% of were of male gender. Overall, 42.2% self-styled as OP. Focusing on the region of origin, 41.7% of participants reported working in Northern Italy, 41.1% in Central Italy, 7.2% in Southern Italy: more precisely and 7.2% were from “Triveneto” regions of Veneto, Trentino-Südtirol and Friuli-Venezia-Giulia, i.e., those encompassing the high-risk area of Alpe Adria. A total of 151 (67.7%) participants acknowledged the diagnosis of cases of human hantavirus cases in Italy as possible, while 10 respondents (4.5%) had reportedly managed cases of hantaviruses in their practice.

### 3.2. Assessment of the Risk Perception

**Figure 1:**
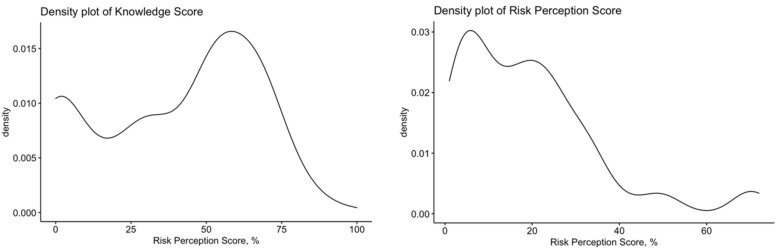
Left to right, density plot for knowledge score, and risk perception score in 223 Italian physicians participating into the survey. Cumulative scores were neither skewed (D’Agostino–Pearson’s normality test *p* value < 0.001 for both scores) nor significantly correlated in Spearman’s ranks correlation test (R = 0.087; *p* = 0.200).

As reported in Table 1, hantavirus infection severity was acknowledged as significant/highly significant by 38.1% of respondents, while only 9.0% of them reported its frequency as significant/highly significant for their daily practice. A correspondent RPS equals to 19.2% ± 16.0 was calculated, whose distribution appeared particularly skewed (D’Agostino–Pearson’s normality test, *p* < 0.001; Figure 1). Focusing on the five occupational groups that were presented to the respondents as potentially at risk for hantavirus infection, 65.5% of them reported a significant/highly significant concern for forestry workers, 64.1% for agricultural workers, 29.6% for construction workers and 18.4% for food processing workers and dairy farmers.

### 3.3. Assessment of Knowledge about Human Hantavirus Infections

Internal consistency coefficient amounted to Cronbach’s alpha = 0.856, suggesting that the resulting score can be acknowledged as reliable, and denoting that a strong relationship exists between the targeted variables. After percent normalization, the mean GKS was relatively low (40.7% ± 40.7; actual range 0–93.3%; median 46.7%), with a skewed distribution, as only 8 out of 223 participants (i.e., 3.6%) reported a score >75.0% (D’Agostino–Pearson normality test *p* value < 0.001; Figure 1). GKS was higher in OP (46.6% ± 25.0) than in other health care providers (36.3% ± 26.2, *p* = 0.011 in independent 2-group Mann–Whitney U Test). In other words, the general understanding of human hantavirus infections was substantially inappropriate, and particularly among non-OP.

In fact (Table 2), the majority of participants exhibited significant knowledge gaps regarding the epidemiology and ecology of hantavirus infections in Italy, including the reported incidence (Q01, 3.6% of correct answers), the actual seroprevalence (Q11, 15.7% correctly reporting 1 to 5%), the higher occurrence in Alpe Adria region (Q12, 31.8%), the estimated case-fatality ratio from European hantaviruses (Q10: 1 to 10%), that was properly reported by only 11.2% of participants, as well as the seasonal trend (Q14, 33.6%).

Interestingly, OP were more frequently aware that rodents are the main hosts of hantaviruses (81.9% vs. 55.8% in other health care providers, *p* < 0.001), rejecting both mosquitos (63.8% vs. 48.1% in other respondents, *p* = 0.028) and ticks as potential vectors for these pathogens (51.1% vs. 32.6%, *p* = 0.008).

Again, clinical characteristics of hantavirus infections were affected by significant misunderstanding. On the one hand, only half of respondents were aware that human hantavirus infections, in the majority of cases, are often overlooked influenza-like illnesses (Q09, 49.8%); on the other hand, about 60.5% of participants were aware that hantaviruses can elicit acute renal failure (Q07), but their role in cases of chronic renal failure was acknowledged by only a quarter of all respondents (Q08, 26.9%).

Focusing on specific signs and symptoms of hantavirus infections (Table 3), the most frequently reported ones were fever (70.0%), proteinuria (64.1%), leukocytosis (62.8%), headache (61.0%), oliguria (i.e., urine flow < 0.5 L in 24 h 57.8%), hematuria (56.1%) and abdominal pain (54.7%). Interestingly, headache (70.2% vs. 54.4%; *p* = 0.023), proteinuria (84.0% vs. 49.6%; *p* = 0.009), hematuria (70.2% vs. 45.7%; *p* = 0.001) and leukocytosis (75.5% vs. 53.5%; *p* = 0.001), as well as signs and symptoms less commonly reported such as polyuria (30.9% vs. 13.2%; *p* = 0.002), and thrombocytopenia (51.1% vs. 32.6%; *p* = 0.008) were more frequently acknowledged by OP than by other health care providers.

### 3.4. Univariate Analysis

RPS and GKS were not correlated (Spearman’s rank correlation test *p* = 0.200). However, as shown in Table 4, medical professionals who acknowledged a higher likelihood for hantavirus infections among agricultural and forestry workers more frequently reported a GKS > median value (78.9% and 81.7%, respectively; *p* < 0.001 in both cases), and were more likely to report the actual occurrence of hantavirus infections in Italy (74.2%, and 75.5%, respectively; *p* < 0.001 in both cases). Similarly, acknowledging higher risk for agricultural and forestry workers not only were mutually associated (*p* < 0.001), but more frequently reported a greater concern for hantavirus infections among food processing workers (85.4% and 90.2%, *p* = 0.003 and *p* < 0.001, respectively) and dairy farmers (87.8% and 97.6%, *p* = 0.001 and *p* < 0.001, respectively). Eventually, acknowledging hantavirus infections as severe ones was more frequently reported by professionals perceiving a higher occupational risk for forestry workers alone (75.5%, *p* = 0.023).

### 3.5. Regression Analysis

In regression analysis (Table 5), outcome variables of perceiving the risk for occupational hantavirus infections as significant/very significant among agricultural workers were assessed through a model that included the following explanatory variables (all of them associated with *p* < 0.25 at univariate analysis): male gender, working as OP, residence in “Triveneto”, acknowledging human hantavirus infections in Italy as “possible”, reporting a better knowledge score, acknowledging human hantavirus infections as severe ones and perceiving a higher risk for occupational infections among forestry workers, dairy farmers, food processing workers and construction workers. Eventually, acknowledging the possible occurrence of hantavirus infections (aOR 21.193, 95%CI 3.666 to 122.505), and the likelihood of occupational infections among forestry workers (aOR 34.993, 95%CI 11.690 to 150.751) and construction workers (aOR 67.915, 95%CI 17.551 to 262.799) were identified as positive effectors.

The regression model assessing the factors associated with a higher perceived risk of hantavirus infection among forestry workers in turn included the following explanatory variables: residence in “Triveneto”, acknowledging human hantavirus infections in Italy as “possible”, reporting a better knowledge score, acknowledging human hantavirus infections as severe ones and perceiving a higher risk for occupational infections among agricultural workers, dairy farmers and food processing workers. Three factors were in turn identified as positive effectors, and namely: higher GKS (aOR 5.880, 95%CI 1.620 to 21.343), higher risk perception for agricultural workers (aOR 33.505, 95%CI 10.995 to 102.103) and higher risk perception for dairy farmers (aOR 26.209, 95%CI 2.516 to 272.936).

## 4. Discussion

The objective of this study was to evaluate KAP on hantavirus infections in a sample of Italian physicians, specifically focusing on OP. More precisely, we assessed the predictors for higher risk perception of hantavirus infections in high-risk groups—i.e., agricultural and forestry workers. Among 223 participants, only 67.7% acknowledged human hantavirus infections in Italy as possible, and less than half of the respondents were aware of the potential severity of these pathogens (38.1%). Even though there is substantial evidence that certain occupational groups (e.g., farmers, forestry workers, etc.) are at greater risk of infection and, also, exhibit higher antibody prevalence than control groups [1,5,9,38,39], only 64.1% and 65.5% of participants acknowledged a higher risk for hantavirus infections among agricultural and forestry workers, respectively.

An appropriate comparison of these data with available evidence is quite difficult. While surveys targeting specific communities and/or occupational groups on knowledge, attitudes and practices for a wide range of health issues (e.g., infectious diseases, vaccine acceptance, etc.) have been successfully used to gather information and planning targeted interventions [29,40,41,42,43], to date only few studies on hantavirus’ KAP have been performed, particularly in European settings [38,44,45,46]. Unfortunately, such studies are limitedly comparable to our estimates, for several reasons. First, all the aforementioned surveys were performed in the Americas, and the participants therefore reported on their understanding of New World hantaviruses rather than on Old World hantaviruses. Second, while we specifically assessed the KAP of medical professionals, the available reports previously focused on residential and occupational groups, of variable health literacy. Eventually, it should be stressed that we specifically stressed the KAP of OP, whose potential importance in the management of hantavirus infections has been recently and strongly addressed [9,47,48], even though their actual understanding of these pathogens and related clinical syndromes largely remains undefined in both high- and middle-income countries.

In fact, our study identified diffuse misunderstandings on the ecology, biology and clinical features of hantavirus infections. This was not unexpected, as there is substantial evidence that medical professionals are often unaware of the symptoms of zoonotic diseases, especially for those that are either rare or perceived as rare ones. As previous studies both on hantavirus [38,46], and from other infectious pathogens [27,29,40], suggest that a better knowledge improves actual preventive practices in targeted population, our estimates may urge for appropriate informative interventions in medical professionals.

As physicians are instrumental in promoting appropriate awareness among the subjects they care for [27,29], these findings support the importance of specific campaigns improving their understanding of hantavirus ecology and clinical features. Without reliable and effective human vaccines, and with treatment options currently limited to the symptomatic care, non-pharmaceutical interventions (NPIs), including standard hygienic practices focusing on rodent control in houses and workplaces, are the cornerstone of our preventive interventions (e.g., seal up holes and gaps; place traps in order to decrease rodent infestations; clean up any easy-to-get food and nesting sites; take precautions when cleaning and/or managing surfaces and/or objects at risk for contamination by rodent fomites) [1,2,38,46,49,50,51].

Moreover, the general overlooking for hantavirus infections may be explained through the inappropriate understanding of these disorders. In fact, RPS and GKS were not significantly correlated, but according to the health belief model, a person’s belief in a health threat, as well as the belief in the effectiveness of the recommended health behavior or action represent the main predictors for the likelihood that the person will adopt the behavior [52]. The large majority of hantavirus infections usually result in a mild disorder, that may be quite simply dismissed as a “summer flu”, alongside several arboviral infections [1,24], while only a small share of total cases develops clinical features that may lead to the eventual diagnosis [1,4,5,6,7,16,53]. In this regard, a critical reappraisal of Italian data may be particularly interesting. On the one hand, to date, autochthonous cases of hantavirus infections have never been officially reported to and by Italian Health Authorities, as recently confirmed by ECDC reports [19,20,21,22]. On the other hand, as recently summarized in a systematic review, since the mid 1980s, various Italian research groups have performed a series of seroprevalence studies on hantavirus in various residential and occupational groups, reporting prevalence rates for IgG that in certain areas were up to 10% of sampled individuals [39]. Moreover, in selected subgroups (i.e., individuals affected by acute kidney diseases), seroprevalence rates were even higher, up to 30% in subjects having a documented exposure to rodents before the onset of the clinical syndrome. In other words, the potential severity of hantavirus infections may have been radically overlooked by most of the respondents, with potential consequences represented by an inappropriate and disproportionately unbalanced trade-off between NPIs and the risk for hantavirus infections, both in health care providers and among their patients, with subsequent consequences in the awareness of the general population.

The inappropriate understanding of hantavirus infections was stressed by the diffuse uncertainties regarding the potential clinical features reported by study participants: even though signs and symptoms associated with the systemic infection and the potential renal involvement (i.e., oliguria, proteinuria, hematuria and leukocytosis) were reported by more than 50% of respondents, the diffuse gaps on other potential features such as hypotension and thrombocytopenia, but also polyuria and back pain hint towards a potential “common sense” answer. In other words, as hantaviruses are often depicted in medical courses, at least in the Italian Core Curriculum for Infectious Diseases, as rare and uncommon pathogens that may cause renal disorders [20,21,22,25,39,54,55], we cannot rule out that a significant share of the respondents might have acknowledged those signs and symptoms that they have perceived as somewhat consistent with the basic information they had. In fact, previous studies performed in the general population potentially exposed to New World hantaviruses had similar or even better understanding of the clinical features of human hantavirus infections [38,46].

Interestingly, OP exhibited a better understanding of hantaviruses than other health care providers. As the share of respondents who reported any previous interaction with hantaviruses was quite low (i.e., a total of 10 respondents, 8 of them OP), a possible explanation may be rather found in the key role played by Italian OP in the health surveillance on the workplaces, and in the higher concern usually paid by OP towards pathogens associated with occupational settings [56,57,58]. Not coincidentally, even in multivariate analysis, the awareness of the potential occurrence of the disorder in the general population, as well as in certain occupational groups, were identified as the main predictors.

*Limitations*. Our study has significant limitations. First, it shares the limits of all Internet-based surveys [40,59,60]. Although reliable, cost-effective and quite a bit faster than a conventional paper-based survey, internet-based surveys are extensively affected by the “self-selection” participants, potentially over-sampling certain sub-groups. Subjects that, because of their better literacy or younger age, are more accustomed to share personal information through the internet access, but also individuals exhibiting a proactive attitude or greater knowledge about the assessed topic, eventually impairing the representativity of the original population. Similarly, the fact of not participating could be understood as a negative attitude or a lack of knowledge about the targeted topic [59]. In this regard, our sample was certainly affected by some degree of self-selection, as suggested by the over-representation of subjects of female gender, of professional from Central Italy while the large majority of all OP are from Northern regions [58], while the representation of the various age groups was satisfyingly consistent with the reference population.

Second, it is reasonable that some of the items assessed through the knowledge test may be affected by a significant social desirability bias, with participants not only reporting “common sense” answers, as previously discussed, but also those answers that may have been perceived as more “appropriate” to fit with the aim of the questionnaire. Therefore, our results could have ultimately overstated the share of individuals having an effective understanding of hantavirus infections associated issues, including the very same knowledge of this disorder [61,62].

Third, despite our sample encompassed medical professionals from the entire country, it could be hardly considered fully representative. In fact, precautionarily assuming the only 50% of participants had some previous knowledge of the pathogen, a I error of 5% (0.05), and power of 95%, a minimum sample size equals to 1.96^2^ × 0.5 × (1 − 0.5)/0.05^2^ = 3.8416 × 0.5 × 0.5/0.0025 = 384 may be calculated, compared to the 233 participants we were able to recruit. On the other hand, as no previous studies on medical professionals have been performed neither in Italy nor in European region, we think that out study may retain a certain significance for health authorities dealing with high-risk areas for hantavirus infections. 

Fourth, even though discussion groups (e.g., by registering only subjects who receive a specific invitation by the manager, answering to specific “selection” questions, etc.) involved in the recruitment of the study participants usually perform a preventive selection, we cannot rule out that some of the respondents did not fully adhere to our selection criteria, furtherly compromising the actual representativity of the sample.

## 5. Conclusions

Our study suggests that Italian Medical Professionals exhibit a certain lack of knowledge on hantavirus-related issues, with an inadequate risk awareness. Interestingly, OP showed a better understanding of these pathogens, as well a more appropriate risk perception for those professions that may result in potential exposure to hantavirus and their hosts. As knowledge status was associated with a more accurate risk perception, at least among certain occupational groups (i.e., forestry workers), it is plausible that filling such information gaps might improve the attitudes of these professionals, and the subsequent spreading of appropriate preventive measures. As hantavirus infections may be effectively countered through effective behavioral practices, improving the specific health literacy of medical professional could be, therefore, instrumental and cost-effective in reducing the potential spreading of such potentially severe infections. 

## Figures and Tables

**Table 1:** Characteristics of 223 Italian physicians participating in the survey (2020). Likert scale for perceived severity and perceived frequency of hantavirus infections were dichotomized as “significant” and “very significant” (i.e., severe and frequently reported disease) vs. all other values (i.e., not severe and infrequently reported).

Variable	No., %	Average ± S.D.
Age (years)		44.2 ± 8.2
Male gender	95, 42.6%	
Working as occupational physician	94, 42.2%	
Residence		
Northern Italy	93, 41.7%	
Central Italy	114, 51.1%	
Southern Italy	16, 7.2%	
Residence in “Triveneto”	16, 7.2%	
Hantavirus infections in Italy acknowledged as possible	151, 67.7%	
Previously managed cases of hantaviruses in the practice	10, 4.5%	
Perceived severity of hantavirus infections in the general population (significant, highly significant)	85, 38.1%	
Perceived frequency of hantavirus infections in the general population (significant, highly significant)	20, 9.0%	
Risk perception score (%)		19.2 ± 16.0
Perceived risk of hantavirus infections (significant, highly significant)		
… among agricultural workers	143, 64.1%	
… among forestry workers	146, 65.5%	
… among construction workers	66, 29.6%	
… among food industry workers	41, 18.4%	
… among dairy farmers	41, 18.4%	
General knowledge score (%)		40.7 ± 26.2
General knowledge score > median (i.e., 46.7%)	109, 48.9%	

**Table 2:** Knowledge test: response distribution of presented items proposed to the 223 medical professionals participating in the survey and contributing to the assessment of general knowledge score (GKS) (Cronbach’s alpha = 0.856). Responses in occupational physicians (OP, No. = 94, 42.2% of total sample) were compared to those of all other health care providers (HPC, No. = 129, 57.8% of total sample) by means of chi squared test (with Yates correction).

Statement	CORRECT ANSWER	TOTAL (No./223, %)	OP (No./94, %)	HCP (No./129, %)	*p* Value *
Q01. In the last five years, human cases of hantavirus infections have been officially reported.	FALSE	8, 3.6%	7,7.4%	1, 0.8%	0.023
Q02. Rodents are the main hosts of hantaviruses.	TRUE	149, 66.8%	77, 81.9%	72, 55.8%	<0.001
Q03. Mosquitos are potential vectors for hantaviruses.	FALSE	122, 54.7%	60, 63.8%	62, 48.1%	0.028
Q04. Ticks are potential vector for hantaviruses.	FALSE	90, 40.4%	48, 51.1%	42, 32.6%	0.008
Q05. Hantaviruses are characterized by frequent interhuman spreading.	FALSE	104, 46.6%	44, 46.8%	60, 46.5%	1.000
Q06. Effective vaccines against hantaviruses are commercially available.	FALSE	116, 52.0%	56, 59.6%%	60, 46.5%	0.073
Q07. Human hantavirus infections can elicit acute renal failure.	TRUE	135, 60.5%	62, 66.0%%	73, 56.6%	0.202
Q08. Human hantavirus infections can elicit chronic renal failure.	TRUE	60, 26.9%	29, 30.9%%	31, 24.0%	0.327
Q09. The majority of human hantavirus infections elicits an Influenza-like illness.	TRUE	111, 49.8%	54, 57.4%%	57, 44.2%	0.069
Q10. Case fatality ratio of European hantavirus infections is estimated …					0.006
<1%	FALSE	25, 11.2%	14, 14.9%%	11, 8.5%	
1–10%	TRUE	25, 11.2%	14, 14.9%%	11, 8.5%	
10–20%	FALSE	9, 4.0%	5, 5.3%	4, 3.1%	
20–30%	FALSE	5, 2.2%	5, 5.3%	0, -	
>30%	FALSE	0, -	0, -	0, -	
Don’t know	-	159, 71.3%	56, 59.6%	103, 79.8%	
Q11. Seroprevalence of hantaviruses in Italian general population is estimated to be …					0.040
<1%	FALSE	45, 20.2%	24, 25.5%	21, 16.3%	
1–5%	TRUE	35, 15.7%	20, 21.3%	15, 11.6%	
5–10%	FALSE	6, 2.7%	5, 5.3%	1, 0.8%	
>10%	FALSE	0, -	0, -	0, -	
Don’t know	-	137, 61.4%	50, 53.2%	87, 67.4%	
Q12. High seroprevalence for hantaviruses in humans and rodents is documented in …					0.006
Alpe Adria region	TRUE	71, 31.8%	35, 37.2%	36, 27.9%	
Western Alps (i.e., Piedmont, Aosta Valley and Lombardy)	FALSE	13, 5.8%	2, 2.1%	11, 8.5%	
Apennine mountains between Tuscany and Emilia-Romagna	FALSE	19, 8.5%	11, 11.7%	8, 6.2%	
Apennine mountains between Umbria and Latium	FALSE	26, 11.7%	6, 6.4%	20, 15.5%	
Po River Valley	FALSE	42, 18.8%	14, 14.9%	28, 21.7%	
Tiber River Valley	FALSE	14, 6.3%	10, 10.6%	4, 3.1%	
Don’t know	-	37, 17.0%	16, 17.0%	21, 16.3%	
Q13. Humans are infected by hantaviruses mainly through …					0.383
Inhalation of aerosols containing urine and feces of infected rodents	TRUE	119, 53.4%	51, 54.3%	58, 45.0%	
Bite of fleas feed up on infected rodents	FALSE	12, 5.4%	7, 7.4%	5, 3.9%	
Bite of infected rodents	FALSE	22, 9.9%	11, 11.7%	11, 8.5%	
Don’t know	-	70, 31.4%	25, 26.6%	45, 34.9%	
Q14. Human hantavirus infections are seasonal ones					0.180
True, peak during the cold season	FALSE	6, 2.7%	1, 1.1%	5, 3.9%	
True, peak during the warm season	TRUE	75, 33.6%	36, 38.3%	39, 30.%	
False	FALSE	29, 13.0%	15, 16.0%	14, 10.9%	
Don’t know	-	113, 50.7%	42, 44.7%	71, 55.0%	
Q15. Official notification of human hantavirus infections is legally required	TRUE	140, 62.8%	69, 73.4%	71, 55.0%	0.008

* chi squared test *p* value.

**Table 3:** Signs and symptoms of hantavirus infections as reported by 223 physicians, broken down as occupational physicians (OP, No. = 94) vs. all other medical health care providers (HCP) participating in the survey. Comparisons were performed by means of chi squared test (with Yates correction).

Clinical Feature	TOTAL (No./223, %)	OP (No./94, %)	HCP (No./129, %)	*p* Value *
Fever (T >38 °C)	156, 70.0%	70, 74.5%	86, 66.7%	0.268
Headache	136, 61.0%	66, 70.2%	70, 54.4%	0.023
Abdominal pain	122, 54.7%	50, 53.2%	72, 55.8%	0.801
Back pain	61, 27.4%	31, 33.0%	30, 23.3%	0.145
Nausea, vomiting	106, 47.5%	40, 42.6%	66, 51.2%	0.256
Petechiae	74, 33.2%	32, 34.0%	42, 32.6%	0.930
Hypotension	49, 22.0%	20, 21.3%	29, 22.5%	0.960
Oliguria (<0.5 L/24 h)	129, 57.8%	94, 100%	35, 27.1%	0.107
Polyuria (>2.0 L/24 h)	46, 20.6%	29, 30.9%	17, 13.2%	0.002
Leukocytosis	140, 62.8%	71, 75.5%	69, 53.5%	0.001
Thrombocytopenia	90, 40.4%	48, 51.1%	42, 32.6%	0.008
Proteinuria	143, 64.1%	79, 84.0%	64, 49.6%	0.009
Hematuria	125, 56.1%	66, 70.2%	59, 45.7%	0.001

* chi squared test *p* value.

**Table 4:** Univariate analysis of factors that in participating health care providers (No. = 223) were associated with higher risk perception for hantavirus infection in agricultural and forestry workers. Comparisons were performed by means of chi squared test (with Yates correction).

Variable	TOTAL (No./223)	Perceived Risk of Hantavirus Infections in Agricultural Workers		Perceived Risk of Hantavirus Infections in Forestry Workers	
		Significant/Very Significant (No.,%)	*p* Value	Significant/Very Significant (No.,%)	*p* Value
Age >50 years	42, 18.8%	28, 66.7%	0.839	29, 69.0%	0.718
Male gender	95, 42.6%	55, 57.9%	0.126	60, 63.2%	0.629
Working as occupational physician	94, 42.2%	55, 58.5%	0.177	64, 68.1%	0.577
Residence in Northern Italy	94, 42.2%	57, 60.6%	0.432	58, 61.7%	0.386
Residence in “Triveneto”	16, 7.2%	14, 87.5%	0.080	14, 87.5%	0.099
Hantavirus infections in Italy acknowledged as possible	151, 67.7%	112, 74.2%	<0.001	114, 75.5%	<0.001
Previously managed cases of hantaviruses in the practice	10, 4.5%	8, 80.0%	0.463	8, 80.0%	0.517
General knowledge score > median (i.e., 46.7%)	109, 48.9%	86, 78.9%	<0.001	89, 81.7%	<0.001
Hantavirus diseases acknowledged as severe (in the general population)	85, 38.1%	60, 70.6%	0.151	64, 75.3%	0.023
Hantavirus diseases acknowledged as frequently reported (in the general population)	20, 9.0%	13, 65.0%	1.000	13, 65.0%	1.000
Perceived risk of hantavirus infections					
among forestry workers	146, 65.5%	125, 85.6%	<0.001	-	-
among agricultural workers	143, 64.1%	-	-	125, 87.4%	<0.001
among construction workers	66, 29.6%	58, 87.9%	<0.001	48, 72.7%	0.186
among food processing workers	41, 18.4%	35, 85.4%	0.003	37, 90.2%	<0.001
among dairy farmers	41, 18.4%	36, 87.8%	0.001	40, 97.6%	<0.001

**Table 5:** Regression analysis of factors that in participating health care providers (No. = 223) were associated with higher risk perception for hantavirus infection of agricultural and forestry workers. Both models included as explanatory factors all dichotomous variables that in univariate analysis were associated with the outcome variables with *p* value < 0.25.

	Perceived Risk of Being Infected by Hantaviruses among Agricultural Workers		Perceived Risk of Being Infected by Hantaviruses among Forestry Workers	
	aOR	95%CI	aOR	95% CI
Male gender	0.585	0.225; 1.525	-	-
Working as occupational physician	1.105	0.290; 3.080	-	-
Residence in “Triveneto”	4.413	0.715; 27.251	0.637	0.068; 5.922
Hantavirus infections in Italy acknowledged as possible	21.193	3.666; 122.505	0.481	0.129; 1.800
General knowledge score >median	0.531	0.134; 2.106	5.880	1.620; 21.343
Hantavirus diseases acknowledged as severe	0.581	0.209; 1.612	1.319	0.511; 3.406
Perceived risk of hantavirus infections				
… among forestry workers	34.993	11.690; 140.751	-	-
… among agricultural workers	-	-	33.505	10.995; 102.103
… among dairy farmers	1.496	0.436; 5.129	26.209	2.516; 272.936
… among food processing workers	1.896	0.533; 6.750	5.219	0.879; 31.000
… among construction workers	67.915	17.551; 262.799	-	-

Notes: aOR = adjusted odds ratio (i.e., odds ratio calculated through binary logistic regression); 95%CI = 95% confidence interval.

## Data Availability

The data presented in this study are available on request from the corresponding author.
